# Relationship between tissue and serum eosinophilia in children undergoing adenotonsillectomy with allergic rhinitis

**DOI:** 10.3906/sag-1904-105

**Published:** 2019-12-16

**Authors:** Nur YÜCEL EKİCİ, Özgür KÜLAHCI

**Affiliations:** 1 Department of Otorhinolaryngology, Adana City Training and Research Hospital, Adana Turkey; 2 Department of Pathology, Adana City Training and Research Hospital, Adana Turkey

**Keywords:** Allergic rhinitis, adenotonsillar hypertrophy, local atopy, tissue eosinophilia, serum eosinophilia

## Abstract

**Background/aim:**

Previous reports suggested that allergic/eosinophilic inflammation affects the adenoid and tonsillar tissue. The aim of this study is to evaluate and compare the tissue and serum eosinophilia in children undergoing adenotonsillectomy with allergic rhinitis.

**Materials and methods:**

The clinical registers of 125 children undergoing adenoidectomy/tonsillectomy due to adenoid/tonsil hypertrophy were examined and reviewed retrospectively. Fifty-seven children with positive skin prick test and symptoms of allergic rhinitis were included in the study as the atopic group, whereas 68 children with no allergic symptoms and negative skin prick test were included as the nonatopic group. Consequently, the total immunoglobulin E level and the serum and tissue eosinophilia of the atopic and nonatopic groups were compared.

**Results:**

Serum eosinophilia in the atopic group was found to be significantly higher than in the nonatopic group (P = 0.045). A significantly higher eosinophil count was found in adenoid/tonsil tissue of the atopic group (P < 0.001, P = 0.023, respectively). However, no significant correlation between tissue and serum eosinophilia was found.

**Conclusion:**

The inconsistency between tissue and serum eosinophilia in atopic children would particularly indicate a role of local atopy in adenotonsillar hypertrophy. Further studies are needed to better understand the effect and usefulness of serum and tissue eosinophilia in children with allergic rhinitis.

## 1. Introduction

Adenotonsillar hypertrophy (ATH) is the most common cause of upper respiratory tract obstruction in childhood. Although the etiology of ATH has not been elucidated, chronic and recurrent inflammatory events around the tissue may be significant because of the decisive role of subject tissues in immune response. Allergy is one of the most common inflammatory processes for the upper respiratory tract [1]. Despite the fact that the prevalence of adenotonsillar disease is reported as 21.2% and 22.9% in some studies [2,3] in children with allergic rhinitis (AR), Griffin et al. [4] reported that the prevalence of AR is similar to age-matched controls. 

Allergic rhinitis is an immunoglobulin E (IgE)-mediated type-1 hypersensitivity reaction of the nasal mucosa which causes eosinophilic inflammation following allergen exposure of the mucous membranes. AR is a clinically defined disease with four main symptoms of rhinorrhea, nasal obstruction, nasal itching, and sneezing. Skin prick tests are widely used to identify the allergen that triggers allergy [5]. The adenoid and tonsil tissues are located at the entrance of the respiratory tract and they function as the first contact points with inhalant allergens. Previous reports suggested that allergic diseases can initiate inflammatory processes affecting the adenoid and tonsil tissues, and that can lead to formation of an allergic inflammation which causes a large number of IgE-positive plasma cells/mast cells, allergen-specific IgE, and eosinophilic infiltration in the tissue [1,6–11]. Having found high numbers of IgE-positive plasma cells in the adenoids of atopic children, Papatziamos et al. [11] argued that lymphatic tissue of adenoids could produce local specific IgE. Another study indicated that locally produced total IgE and specific IgE antibodies to *Dermatophagoides pteronyssinus* in adenoid tissue homogenate are significantly higher in atopic children than in nonatopic, and they reported that these antibodies may lead to eosinophilic infiltration in adenoid tissue of atopic children [9]. The aforementioned studies proposed that allergic/eosinophilic inflammation occurs in adenoid and tonsil tissue of children with AR. Recent allergy studies suggested that a localized allergic response might occur without systemic atopy in adenoid and tonsil tissue [8,12]. In our previous study, we investigated the number of eosinophils in adenoid and tonsil tissue of sensitized children, and we have found that the AR diagnosis may be supported by tissue eosinophilia [13]. The current study hereby aims to evaluate the serum and tissue eosinophilia in children with ATH and investigate the correlation of serum and tissue eosinophilia in children with AR.

## 2. Materials and methods

### 2.1. Study population

Approval from the Ethics Committee of Adana Teaching and Research Hospital was obtained for the study (Ethics Committee No./Date: 168 / 28.Feb.2018). Between March 2017 and February 2018, we reviewed and investigated the clinical registers of 125 children undergoing adenoidectomy/tonsillectomy due to ATH in the Department of Otorhinolaryngology, Adana City Training and Research Hospital. In the patient registry files, sex, age, detailed histories of systemic disease, clinical visit notes, results of allergic tests (skin prick test and serum total IgE), and complete blood cell count results within 1 week prior to surgery of patients were evaluated. The patients with asthma, immunodeficiency, autoimmune diseases, drug-induced diseases, infectious diseases, cranial or genetic syndromes, vitamin D deficiency, and insufficient file information were excluded from the study. The severity of adenoid and tonsil hypertrophy was classified in accordance with the Brodsky criteria [14]. Surgical indications were ≥grade 3 tonsil growth and adenoids that cause obstructive sleep apnea. A total of 125 patients between 3 and 10 years old were included in the study. The children with clinical symptoms of rhinorrhea, nasal obstruction, nasal itching, and sneezing and also positive skin test were assigned to the atopic group (n = 57), while the children with no clinical symptoms and negative skin test were assigned to the nonatopic group (n = 68). 

### 2.2. Skin prick test 

Before the surgery, skin prick tests were performed with a multitest applicator for the thirty most common aeroallergens by standard Alyostal ST-IR (Stallergenes SA, France) allergen extracts (Table 1). Before application of the skin prick test, parents of children were questioned about the status of drug use (antihistamines, antitussives, corticosteroids, H2 receptor antagonists) in the past 7 days. Histamine hydrochloride (10 mg/mL) and physiological saline were used as positive and negative controls, respectively. Skin reactions were measured 20 min immediately after the application, and skin induration with ≥3 mm diameter or larger than the negative control was considered as a positive reaction. 

**Table 1:** Allergen sensitization.

Allergen panel	Sensitized group, (n/total) %
Dermatophagoides farinae	71.93 (41/57)
Dermatophagoides pteronyssinus	63.16 (36/57)
Betulaceae (Betula alba, Alnus glutinosa, Carpinus betulus, Corylus avellana)	-
Salicaceae (Populus alba, Salix caprea)	3.51 (2/57)
Mixture of 12 grasses (Lolium perenne, Dactylis glomerata, Phleum pratense, Anthoxanthum odoratum, Poa pratensis, Festuca elatior, Agrostis vulgaris, Holcus lanatus, Cynodon dactylon, Avena sativa, Avena fatua, Lotus corniculatus)	8.77 (5/57)
Oleaceae (Olea europaea, Ligustrum vulgare, Fraxinus excelsior)	-
Compositae (Solidago candensis, Taraxacum officinale, Chrysanthemum leucanthemum, Xanthium)	-
Aspergillus mix (Aspergillus fumigatus, Aspergillus niger, Aspergillus nidulans)	3.51 (2/57)

### 2.3. Pathological examination

The adenoid and tonsil tissue samples taken in the operation were examined with microscopy in hematoxylin and eosin-stained sections. Sections were scored under 400× magnification in a blinded fashion by the same pathologist. Eosinophils were counted in 10 random sections for all tissue samples.

### 2.4. Complete blood cell counts

Blood samples were obtained within one week prior to surgery. The eosinophil count (103 µL) was examined with a fully automated cell counter (Sysmex XN-9100 Automated Hematology System, Kobe, Japan). 

### 2.5. Statistical analysis

The Shapiro–Wilk test was performed to test the suitability of the normal distribution of numerical data. Descriptive analyses were presented using means ± standard deviations (SD) for normally distributed variables. Independent sample t-tests were used for parametric variables comparison. The chi-square test was used for the relationships between categorical variables. The correlation coefficient was calculated to investigate the conformity between two evaluation criteria in the categorical variables. ROC curve analysis was performed to find the cutoff value for variables to predict the development of sensitivity. For that, sensitivity, specificity, and area under the curve were calculated. P < 0.05 was considered to be statistically significant. 

## 3. Results

Demographic characteristics of 125 children between 3 and 10 years old included in the study were summarized and are exhibited in Table 2. There was no significant difference between atopic and nonatopic groups in the terms of sex and age distribution (P = 0.488 and P = 0.259, respectively).

**Table 2:** Demographic variables of the groups.

Variables	Atopic group	Nonatopic group	P
Sex			0.488
Boys, n	33 / 57	40 / 68	
Girls, n	24 / 57	28 / 68	
Age, mean ± SD, years	6.394 ± 2.207	5.786 ± 2.364	0.259
Total IgE, mean ± SD	2.538 ± 0.471	2.355 ± 0.538	0.139**
Tissue eosinophilia			
Adenoid, mean ± SD	0.798 ± 0.293	0.378 ± 0.336	<0.001**
Tonsil, mean ± SD	0.472 ± 0.257	0.237 ± 0.321	0.023**
Serum measurements			
Eosinophils, mean ± SD, ×10^3^ µL	0.416 ± 0.342	0.269 ± 0.283	0.045*

IgE: Immunoglobulin E *P: Independent sample t-test **P: log transformation

In serum total IgE levels, no significance between atopic and nonatopic groups (P = 0.139) existed. There were 41 adenotonsillectomies and 16 adenoidectomies in the atopic group and 45 adenotonsillectomies and 23 adenoidectomies in the nonatopic group. Totally 125 adenoid (57 adenoid from atopic group, 68 adenoid from nonatopic group) and 86 tonsil (41 from atopic group, 45 from nonatopic group) tissue samples were examined on the basis of the number of eosinophils. There was a statistically significant difference between atopic and nonatopic groups with respect to tissue eosinophilia (P < 0.001 for adenoid tissue, P = 0.023 for tonsil tissue) (Table 2). Serum eosinophil counts were significantly higher in the atopic group (P = 0.045). A significant correlation was found between the number of eosinophils in adenoid and tonsil tissues of children undergoing adenotonsillectomy operation (r = 0.676, P < 0.001). On the other hand, there was no significant correlation between tissue eosinophilia and serum eosinophilia (r = 0.064 and P = 0.588 for adenoid and serum eosinophilia; r = 0.017 and P = 0.906 for tonsil and serum eosinophilia) (Table 3). 

**Table 3:** Correlation of variables between tissue and serum.

Variables	Study group; n	Correlation coefficient; r	P-value
Correlation of tonsil and adenoid eosinophilia	86	0.676	<0.001**
Correlation of adenoid eosinophilia and serum eosinophilia	125	0.064	0.588
Correlation of tonsil eosinophilia and serum eosinophilia	86	0.017	0.906

Pearson’s correlation coefficient (rho) **Correlation is significant at the 0.01 level (2-tailed).

In order to find the optimal value of tissue eosinophilia and serum eosinophil count, ROC analysis was performed. Associated results are depicted in Table 4. The predictive value of eosinophil in the tissue was found to be >4/10 high-powered fields for adenoid and >2/10 high-powered fields for tonsil tissue. Sensitivity and specificity value of these cutoff points were found as 75.8%–88.1% for adenoid tissue and as 56.5%–92.9% for tonsil tissue, respectively (P = 0.0001). The predictive value of eosinophil in the serum was >0.345 × 103 µL for eosinophil count (sensitivity value: 54.5 and specificity value: 78.6; P = 0.028). Sensitivity and specificity of tissue eosinophilia were found to be higher than those of serum eosinophilia, particularly in adenoid tissue.

**Table 4:** Results of ROC analysis.

Variables	Cutoff value	AUC	Sensitivity	Specificity	P-value
Adenoid eosinophilia	>4	0.816	75.8	88.1	0.0001
Tonsil eosinophilia	>2	0.786	56.5	92.9	0.0001
Serum eosinophilia, ×103 µL	>0.345	0.649	54.5	78.6	0.028

AUC: Area under the curve

The most common allergens were found to be *Dermatophagoides farinae* [71.93% (41/57)] and *Dermatophagoides pteronyssinus* [63.16% (36/57)] (Table 1). Fifteen children demonstrated single sensitization, whereas 42 children had polysensitization. No conspicuous association was distinguished between the polysensitization and single sensitization groups with respect to adenoid eosinophilia, tonsil eosinophilia, and serum eosinophilia (P = 0.699, P = 0.794, P = 0.208, respectively). 

## 4. Discussion

Several authors suggested that allergic/eosinophilic inflammation affects the homeostasis of adenotonsillar tissue. Increased expression of IgE-positive cells/specific IgE antibodies, CD1a+ Langerhans cells, and IL-4 and IL-5 mRNA-positive cells was indicated in adenoid tissue of children with AR [6,9–11]. Recently, the pathophysiology of local allergic rhinitis (LAR) was defined as presence of allergen-specific IgE in the nasal mucosa in the absence of skin prick test and serum specific IgE test positivity in patients with symptoms of allergic rhinitis [15,16]. LAR is known as a localized nasal allergic response without systemic atopy. After exposure to aeroallergens, nasal Th2 IgE-mediated inflammation occurs in the tissue. Eosinophil cationic protein, eosinophils, mast cells, basophils, CD3+ T cells, and CD4+ T cells increase in the tissue [16]. LAR can be identified with multiple nasal provocation tests, locally produced specific IgE, or other local inflammation markers of allergic response. Most recently, allergy studies began to draw attention to local atopy in children with ATH [7,9,11]. Correspondingly, Zhang et al. [12] compared the sensitization type of serum and local tissue in children with ATH. Among 20 children with ATH, specific IgE is locally produced in both adenoid and tonsil tissue homogenate, but almost half of them were found to be negative for serum specific IgE. Cho et al. [8] demonstrated that 68.6% of children with ATH were sensitized to more than one allergen in adenoid and tonsil tissues, and 53.9% of children with ATH were sensitized to serum. Furthermore, 36.2% of children with specific IgE negativity in the serum had positive specific IgE in adenoid and tonsil tissue. Also, they reported more severe nasal symptoms in children with local atopy [8]. Discrepancy between adenoid/tonsil tissue and serum specific IgE may indicate that local allergic inflammation plays an important role in ATH [8,12].

Due to the primary effector cells of allergic diseases, eosinophils are often found to be increased in serum and related tissues. We have previously reported that the number of eosinophils in tissues of sensitized children undergoing adenotonsillectomy due to hypertrophy and/or recurrent infection was significantly higher and that the presence of eosinophils in ≥5/10 high-powered fields in adenoid tissue and ≥3/10 high-powered fields in tonsil tissue may indicate sensitization [13]. The present study finds similar results in children with ATH. It must be noted that findings about the relationship between serum eosinophilia and AR are conflicting in the literature. For instance, while Chen et al. [17] suggested that serum eosinophil count supports the diagnosis and prediction of the severity of AR, Yenigün et al. [18] found a relationship between serum eosinophilia and AR in children with positive skin prick test results. However, the latter did not find any significant relationship with the symptomatology of allergic rhinitis. These reports may indicate that allergic response is not only limited to local inflammation, but it also causes an increase in systemic inflammation. Reasonably, locally produced specific IgE might contribute to serum eosinophilia. 

In our study, both tissue eosinophilia (adenoid and tonsil) and serum eosinophilia were found to be higher in atopic children. However, there was no statistically significant relationship between tissue and serum eosinophilia. This inconsistency may support the presence of local atopy in childhood ATH. Additionally, we found that eosinophilia in the tonsil and adenoid tissue has a significant correlation (P < 0.001, correlation coefficient: 0.676). This result may be interpreted by the anatomical proximity of these tissues to each other. 

According to ROC analysis, the cutoff value of serum eosinophil count was >0.345 × 103 µL (sensitivity value: 54.5%, specificity value: 78.6%) in the atopic group. As depicted in this study, the sensitivity and specificity of tissue eosinophilia were higher than those of serum eosinophilia. Although all these findings demonstrated the diagnostic significance of serum eosinophilia in AR, serum eosinophilia is still less diagnostic in itself. This is because, apart from allergic diseases, it is known that immune deficiencies, autoimmune diseases, drug-induced diseases, and infectious diseases (especially parasitic helminth infections) are also associated with serum eosinophilia [19]. 

As in previous studies, in our study, the most common allergens detected in skin prick test results were *Dermatophagoides farinae* and *Dermatophagoides pteronyssinus* [8,13,19]. Forty-two children (73.68%) had polysensitization and 15 children (26.32%) had single sensitization. Congruent with previous literature, polysensitization was found to be more common in children with ATH [3]. Because of the high polysensitization ratio and small size of our study group, we did not perform statistical analyses to determine which allergen is more eosinophilic. However, we found that polysensitization was not more significant than single sensitization on the basis of tissue and serum eosinophilia. 

Although it has been reported in the literature that local allergic inflammation may play a role in ATH and cause allergic respiratory symptoms in children, limited data are available about the clinical implications and management modalities of local allergic rhinitis in these patients [1,8,12]. It may develop into systemic classical allergic disease or these patients may remain stable over a long period of time. Further randomized clinical studies are needed to obtain more reliable results on this important clinical issue. 

Among the limitations of our study, it might be stated that it was conducted at a single center due to lack of symptomatology of clinical allergy, and that it has a retrospective character. Additionally, the study population was relatively small, and solely inhaled allergens were evaluated. Further studies are required to describe the value of eosinophilia in tissue and serum, such as large prospective series and profiles with cytokine, specific IgE, and other allergic mediators.

To the best of our knowledge, this was the first study to demonstrate no correlation between serum and tissue eosinophilia in children with AR. The discrepancy in eosinophilia between tissue and serum may indicate a role of local atopy in adenoid and tonsil tissue. At the same time, tissue eosinophilia may be utilized for the diagnosis of LAR in children undergoing adenotonsillectomy due to ATH. Examination of tonsil and adenoid specimens in terms of eosinophilia is a simple and cost-effective method.
